# Improving diagnostic and management competencies for common orofacial conditions among new dental graduates: the effect of an educational intervention

**DOI:** 10.1186/s12903-026-07662-7

**Published:** 2026-01-12

**Authors:** Suad Aljohani, Sana Alhamed, Arwa M Farag, Salha R Aljohani, Ahoud Jazzar, Hebah AlDehlawi, Abdullah Almohammadi, Malik Alghamdi, Khalid Aljohani

**Affiliations:** 1 Department of Oral Diagnostic Sciences, Faculty of Dentistry, King Abdulaziz University, Jeddah, Saudi Arabia; 2 Department of Orthodontics, Faculty of Dentistry, King Abdulaziz University, Jeddah, Saudi Arabia; 3 Faculty of Dentistry, King Abdulaziz University, Jeddah, Saudi Arabia

**Keywords:** Common orofacial conditions, Dental training programs, Management of orofacial diseases, Oral diagnosis, Dental interns, Oral lesions

## Abstract

**Background:**

The early and accurate diagnosis and management of common orofacial conditions is essential for effective dental care. However, junior dentists often lack confidence and practical skills in this area. This study aimed to assess the impact of an educational lecture on dental interns’ competency in diagnosing and managing prevalent orofacial diseases.

**Methods:**

This study was conducted among dental interns at King Abdulaziz University, Saudi Arabia. A total of 161 newly graduated interns were invited to participate. An evidence-based, one-hour interactive online lecture involving common orofacial conditions was delivered by a certified oral medicine consultant. Participants completed a validated 40-item questionnaire at three time points: before the lecture, immediately after the lecture, and two months after the lecture. The questionnaire was designed to assess diagnostic and management skills using 20 clinical scenarios. Diagnostic competency score was based on the percentage of correct responses to 20 diagnostic multiple-choice questions (MCQs), and a management competency score was based on the percentage of correct responses to 20 management MCQs. Descriptive statistics and weighted kappa analyses were conducted by employing SPSS^®^, with significance set at *p* < 0.05.

**Results:**

Among the 161 participants, 91 interns (56.5%) completed all the stages. Following the intervention, overall competency scores significantly increased (from 78% at baseline to 91% immediately after the lecture), with a relative improvement of approximately 13% being observed (*p* < 0.001); moreover, the scores remained 8.5% higher than those at baseline at the two-month evaluation. Diagnostic accuracy demonstrated a notable improvement of 12.5% (*p* < 0.001) following the intervention and remained higher than baseline at follow-up. The greatest improvement in the diagnosis was observed for herpes labialis (+ 53.1%), irritational fibroma (+ 32.1%), and leukoedema/morsicatio buccarum (+ 20%). Management competency improved by 13.5% (*p* < 0.001) immediately after the lecture, followed by a decrease of 6% at the two-month follow-up. The greatest improvement was observed in the management of recurrent aphthous stomatitis (+ 36.6%). No significant differences were observed in terms of sex or grade point average (GPA).

**Conclusion:**

The educational lecture significantly enhanced the ability of dental interns to diagnose and manage common orofacial conditions. Given the importance of timely diagnosis and appropriate management in reducing the severity of common oral lesions, the findings of this study provide valuable insights. The results suggest that such targeted educational interventions should be integrated into continuing dental education programs to enhance clinical competence among practitioners.

**Supplementary Information:**

The online version contains supplementary material available at 10.1186/s12903-026-07662-7.

## Introduction

Orofacial conditions are diverse and can arise from various causes. A significant challenge in the oral cavity is that different conditions may lead to similar clinical signs and symptoms. This can make it difficult for nonexperts to accurately diagnose or evaluate such findings, thus potentially resulting in delays in diagnosis and management. The prognosis of the specific orofacial condition may be compromised by such delays [1]. Many oral lesions typically require no treatment and are primarily diagnosed through clinical examination and detailed patient history taking [2]. However, if a dentist with limited experience encounters these findings, undue stress may be inflicted on the patient, which can lead to unnecessary investigations and/or treatments, thus ultimately increasing costs for the patient and the health system [3]. In contrast, some oral findings may necessitate additional investigations to rule out more serious conditions. To provide appropriate management for different orofacial conditions, it is essential to establish an appropriate diagnosis and understand the causative risk factors, associated signs and symptoms, and available treatment options. Furthermore, the education of patients regarding their oral conditions and the implementation of appropriate management strategies can help to minimize the severity of common oral lesions [4–6].

General dental practitioners (GDPs) play a crucial role in identifying common oral lesions during their daily practice, as they may be the first healthcare providers to detect these conditions [7, 8]. The management of these lesions typically falls within the knowledge and competencies expected from the GDPs, as reflected in the undergraduate training curriculum. Moreover, referral to oral medicine specialists may be impractical in rural areas. Therefore, it would be beneficial to enrich GDPs with the knowledge and skills that are necessary to recognize and manage common oral lesions that are observed within their scope of practice [8–10].

The ability of dental students and GDPs to detect and diagnose oral mucosal lesions has been previously assessed [9–14]. A survey conducted in Finland assessed the level of self-assessed competency among undergraduate dental students in oral mucosal lesions diagnosis [13]. The results revealed that approximately 51% of the students did not believe that their knowledge adequately met the needs for effective management. Consequently, educational improvement was recommended. Another survey was conducted in Saudi Arabia to assess senior dental students’ abilities to diagnose and manage common oral lesions, which revealed that while 94% of the students were able to correctly diagnose clinical cases, only 78.5% were able to correctly manage the cases [8]. This study revealed that there is a critical need to enhance the knowledge of dentists concerning the diagnosis and management of oral mucosal lesions.

The current study was performed with the aim of assessing the baseline knowledge of recent dental graduates and evaluating the impact of a concise, well-structured educational lecture designed to enhance their competency in diagnosing and managing common orofacial conditions, thus ultimately contributing to improved patient outcomes and satisfaction. Although short-term educational interventions can substantially improve diagnostic and management skills, these gains may not be sustained without periodic reinforcement. Therefore, continuous educational efforts are essential for consolidating learning and preventing the gradual decline of knowledge over time [3, 15, 16]. Despite extensive undergraduate training, many junior dentists still exhibit gaps in diagnostic confidence and management decision-making for common orofacial conditions. Few studies in Saudi Arabia have evaluated targeted educational interventions addressing these gaps. This study focused on enhancing the ability to detect and manage common oral lesions, thereby contributing to improved patient care and overall quality of life. The early recognition and appropriate management of oral lesions are critical competencies for dental practitioners, as these conditions can often serve as early indicators of systemic disease or potentially malignant disorders. The findings of this study highlight the importance of continuous professional development beyond undergraduate education. Furthermore, this study investigated whether a structured educational intervention implemented after graduation can effectively bridge the gap between theoretical learning and independent clinical practice, thereby reinforcing clinical decision-making skills and promoting confidence in the diagnosis and management of oral lesions.

## Materials and methods

This study was conducted among dental interns at King Abdulaziz University (KAU) in Jeddah, Saudi Arabia. This study was approved by the research ethical committee of the faculty of Dentistry at King Abdulaziz University (174-11-23). All of the participants signed an informed consent form before they participated in all of the stages of the study, with the freedom to withdraw from the study at any time. The study intended to include all KAU dental interns for the academic year 2023/2024, ultimately comprising a total of 161 participants (86 females and 75 males). In Saudi Arabia, dental interns are recent graduates who are completing a one-year mandatory clinical training (under supervision) before obtaining full licensures as general dental practitioners (GDPs). During this internship, they manage patients independently under faculty supervision across various dental specialties.

### Hypothesis

The delivery of a short, evidence-based, interactive lecture will significantly improve dental interns’ diagnostic and management competencies for common orofacial conditions, which would be sustained over a short-term follow-up period.

### Importance of the study

The enhancement of diagnostic and management performance reduces unnecessary referrals, improves patient outcomes, and supports continuing education development.

### The educational lecture

The lecture involved a one-hour session delivered by a certified oral medicine consultant. It was conducted online via Google Meet using PowerPoint and included an interactive component that encouraged engagement between the dental interns and the consultant, thus simulating a traditional learning environment. The scientific content was based on the best available evidence, and the common orofacial lesions were derived from published articles relevant to Saudi Arabia [6, 7, 17].

The lecture covered common orofacial conditions and detailed their clinical findings, diagnosis, prevalence, and management strategies. The lecture addressed twenty commonly encountered orofacial lesions, including recurrent aphthous stomatitis, herpes labialis, irritational fibroma, traumatic ulcer, geographic tongue, fissured tongue, hairy tongue, leukoedema, oral lichen planus, mucocele, Fordyce granules, nicotinic stomatitis, lingual varices, ankyloglossia, morsicatio buccarum, atrophic glossitis, physiologic pigmentation, pyogenic granuloma, torus palatinus, and scalloped tongue. The one-hour online lecture was conducted via Google Meet using case-based slides, clinical photographs, and open question and answer discussions.

### The questionnaire

**Fig. 1:**
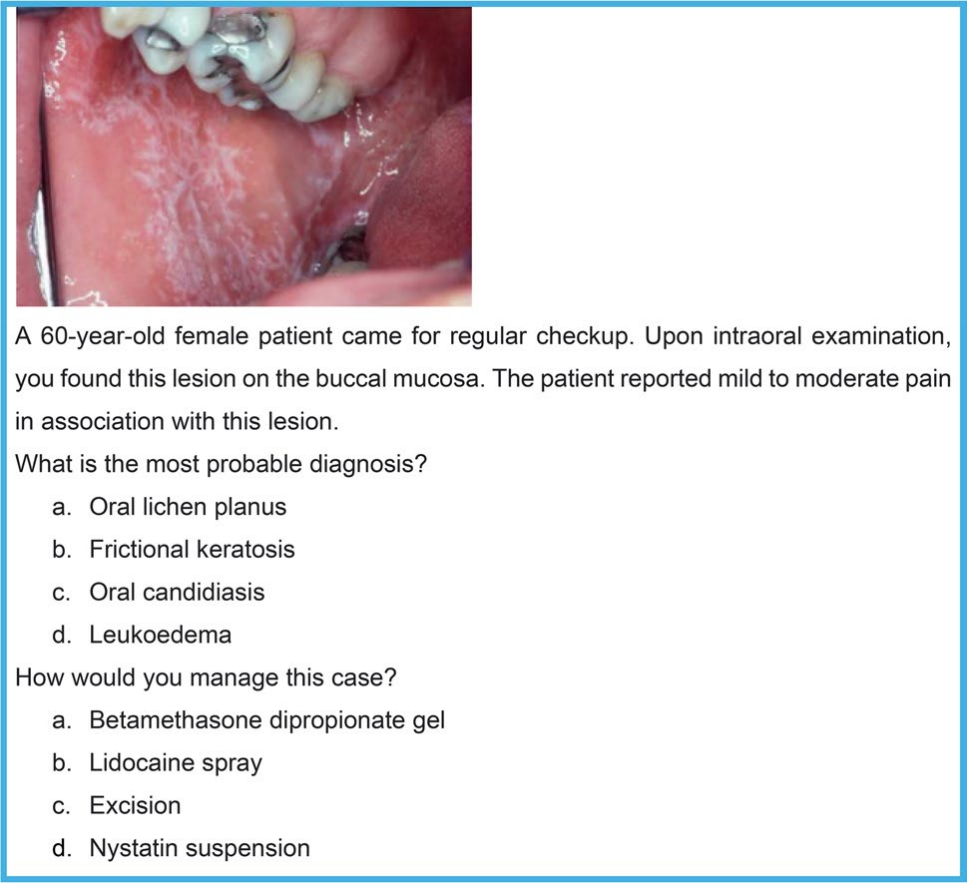
Case-based scenario with a clinical image and two multiple-choice questions used to assess diagnostic and management competencies in the questionnaire

An online questionnaire was designed in Google Forms. The form began with a consent statement to ensure voluntary participation in the study before the participants proceeded to the survey questions. The questionnaire consisted of 40 multiple-choice questions (MCQs) based on 20 clinical scenarios; moreover, two questions were formulated for each scenario, with the first question focusing on diagnosis, and the second question focusing on management (Fig. 1). The case scenarios were carefully designed to include keywords that were relevant to the characteristics of the specific oral condition, accompanied by a clear representative picture (Supplementary File 1).

### Validity and reliability

The content of the questionnaire was reviewed by four oral medicine consultants for validation. To assess questionnaire reliability, it was administered to three subjects who were excluded from the main sample. The same questionnaire was readministered to these subjects after one week. The agreement between the responses from the two occasions was evaluated using the weighted kappa statistic, which was found to be 0.937 (95% CI, 0.877–0.996), indicating excellent reliability of the questionnaire. A small pilot group (*n* = 3) was used for test-retest reliability to ensure feasibility and to minimize participant fatigue before full data collection. The 40-item questionnaire was validated by four oral medicine consultants for content and face validity. Test-retest reliability on three pilot subjects produced a weighted κ = 0.937 (95% CI: 0.877–0.996).

### Statistical analysis

Statistical analyses were performed by using IBM SPSS Statistics^®^ version 29.0.0 (IBM Corp., Armonk, NY, USA). Descriptive statistics in the form of percentages of correct answers were calculated for categorical variables and means; additionally, standard deviations were calculated for continuous variables. The dependent variables included diagnostic competency scores (percentages of correct diagnosis responses) and management competency scores (percentages of correct management responses), which were calculated from 40 MCQs (including 20 diagnostic and 20 management questions) derived from 20 clinical scenarios. Each dental intern’s overall competency score represented the mean percentage across all of the items and was assessed at three timepoints, including pre-intervention (baseline), immediately post-intervention, and at the two-month follow-up. The independent variables were sex (male/female) and GPA category (A, B, C), which were used to compare mean changes in competency scores across groups.

In this study, diagnostic competency was defined as the ability of dental interns to accurately identify and differentiate oral mucosal lesions by integrating patient history, clinical examination findings, and the formulation of differential diagnoses. This definition aligns with the cognitive and analytical domains of clinical reasoning emphasized in international dental education frameworks, including those of the Association for Dental Education in Europe (ADEE) and the American Dental Education Association (ADEA) [18–21]. Similarly, management competency was defined as the ability to develop and justify an appropriate management plan for a diagnosed condition, encompassing patient education, selection of treatment or referral options, and application of evidence-based principles in clinical decision-making [19, 22].

The GPA in the Saudi grading system ranges from 0.0 to 5.0 and was converted to alphabetical grades (A, B, C) for descriptive purposes. An A grade generally represents excellent performance and corresponds to a GPA between 3.7 and 5.0 (or a percentage of approximately 90–100%). A B grade reflects good or above-average performance, which is usually equivalent to a GPA range of 2.7 to 3.6 (or approximately 80–89%). A C grade indicates satisfactory or average performance and corresponds to a GPA between 1.7 and 2.6 (or approximately 70–79%).

To compare the total questionnaire scores between the timepoints of pre-intervention, immediately post-intervention, and the 2-month follow-up, a related-samples Friedman’s two-way analysis of variance by ranks was used, followed by post hoc pairwise comparison. Afterward, the changes in questionnaire scores between the pre-intervention and immediately post-intervention timepoints were calculated, in addition to the changes in scores ranging from pre-intervention to 2 months after the intervention. The mean change or difference was compared between sex and GPA categories by using the independent-samples Mann-Whitney U test for assessing differences between males and females and the independent-samples Kruskal-Wallis test for assessing differences between the three categories of GPA. Weighted kappa was solely applied to assess test-retest reliability.

## Results

A total of 161 dental interns participated in the first stage of the questionnaire. However, only 91 dental interns (62 males and 29 females) completed all three stages, thus resulting in a response rate of 56.5%. Seventy interns did not complete all three stages of the study. Comparisons of demographic characteristics (including sex and GPA) revealed no statistically significant differences between those who completed all stages and those who discontinued participation.

Table 1 displays a comparative analysis of the questionnaire scores prior to the lecture, after the lecture, and two months following the lecture. The scores for the questions were categorized into diagnosis and management scores, as well as a total score. The diagnosis and management scores were significantly higher after the lecture and at two months after the lecture (*p* < 0.001) in comparison to the values recorded before the lecture. The average total score of correct responses before the lecture was 78 ± 16.5%, which was significantly lower than the scores obtained immediately after the lecture (91 ± 8%) and at two months after the lecture (86.5 ± 11.3%). Post hoc analysis indicated that the overall score obtained immediately after the lecture did not significantly differ from the score obtained two months after the lecture (*p* = 0.083).

**Table 1:** The average questionnaire scores at baseline, post-lecture, and two-month follow-up across the assessment periods. *Statistically significant (*p <* 0.05)

Category	Before lecture(1)	After lecture(2)	2 months after (3)	Change	Change	*p* value		
				1 vs. 2	1 vs. 3	1 vs. 2	1 vs. 3	2 vs. 3
Diagnosis (%)	81.5 ± 17	94 ± 6	91 ± 9	+ 12.5	+ 9.5	< 0.001*	0.004*	0.225
Management (%)	74.5 ± 17	88 ± 8.5	82 ± 12.5	+ 13.5	+ 7.5	< 0.001*	< 0.001*	0.909
Total (%)	78 ± 16.5	91 ± 8	86.5 ± 11	+ 13	+ 8.5	< 0.001*	< 0.001*	0.083

### Diagnostic competency

The baseline diagnostic accuracy was 81.5 ± 17%. Following the lecture, this accuracy increased by 12.5%, with a slight decline being observed after two months (Table 1).

The diagnostic performance of dental interns in identifying common orofacial diseases markedly improved after the educational intervention, as summarized in Table 2. The greatest increase was observed in the detection of herpes labialis, with a 53.1% increase being observed immediately after the lecture. Moreover, a 32.1% improvement was observed for the diagnosis of irritational fibroma. Comparable progress of 20% was noted for leukodema and morsicatio buccarum. The diagnostic accuracy for recurrent aphthous stomatitis, atrophic glossitis, and traumatic ulcers was observed at 100% after the lecture, with this accuracy being maintained with minimal or no reduction at the two-month follow-up.

**Table 2:** The percentage of correctly answered questions across the three questionnaires

Oral Lesion	Diagnosis			Management			
	Before (%)	After (%)	2 months (%)	Before (%)	After (%)	2 months (%)	
Nicotinic stomatitis	81.3	98.2	91.2	81.3	94.7		86
Recurrent aphthous stomatitis	82.4	100	87.7	38.5	75.1		54.4
Oral lichen planus	75.8	87.7	77.2	45.1	49.1		40.4
Herpes labialis	45.1	98.2	96.5	86.8	96.5		87.7
Ankyloglossia	81.3	89.5	84.2	94.5	89.5		82.5
Scalloped tongue	82.4	98.2	93	70.3	89.5		82.5
Irritational fibroma	36.3	68.4	68.4	90.1	94.7		89.5
Geographic tongue	92.3	94.7	94.7	14.3	28.1		10.5
Lingual varices	91.2	98.2	98.2	68.1	89.5		91.2
Mucocele	82.4	94.7	96.5	85.7	98.2		94.7
Pyogenic granuloma	73.6	86	91.2	83.5	91.2		87.7
Atrophic glossitis	89	100	96.5	89	98.2		96.5
Leukoedema	72.5	96.5	91.2	84.6	98.2		93
Traumatic ulcer	91.2	100	94.7	84.6	98.2		96.5
Fissured tongue	89	96.5	96.5	69.2	91.2		87.7
Torus palatinus	90.1	96.5	93	72.5	91.2		86
Morsicatio buccarum	67	87.7	87.7	90.1	98.2		96.5
Hairy tongue	93.4	98.2	98.2	80.2	96.5		91.2
Physiologic pigmentation	76.9	91.2	91.2	84.6	94.7		91.2
Fordyce granules	89	96.5	94.7	85.7	98.2		98.2

### Management competency

A parallel increase in management ability of 13.5% was observed (from 74.5 ± 17% to 88 ± 8.5%), with a decline to 82% being observed upon retention evaluation.

The greatest improvement in management ability was observed for recurrent aphthous stomatitis, with an increase from 38.5% to 75.1% being observed immediately after the lecture; however, a decline to 54.4% was observed after 2 months. Other conditions that demonstrated marked improvement included a fissured tongue (22%) and lingual varices (21.4%). Moderate improvement was observed in conditions such as a hairy tongue (16.3%) and both leukoedema and traumatic ulcers (both 13.6%). In contrast, oral lichen planus and irritational fibroma demonstrated only a minimal enhancement of 4%.

Prescription competency was determined to be 46.1% at baseline and improved to 62.2% immediately after the lecture; however, it returned to nearly the same baseline level of 48.6% during follow-up.

### Correlation with sex and GPA

When comparing changes in knowledge score by sex, compared with females, males demonstrated greater increases both immediately after the lecture and at the two-month follow-up; however, these differences were not statistically significant (*p* = 0.488 and *p* = 0.621, respectively). With respect to GPA, students with a B grade demonstrated the greatest increase in scores, followed by those with a C grade, whereas students with an A grade demonstrated the smallest change. Nonetheless, the observed differences among GPA groups were not statistically significant, before to after lecture and before to 2 months after lecture (*p* = 0.391 and *p* = 0.210, respectively) (Table 3).

**Table 3:** Improvement in scores according to sex and GPA

		Improvement (before to after lecture)	Improvement (before to 2 months after lecture)
Gender	Male	5.4 ± 6.2	3.7 ± 5.8
	Female	3.8 ± 5.6	2.8 ± 5.8
	*p* value †	0.488	0.621
GPA	A	3.7 ± 5.2	3.1 ± 4.5
	B	6.3 ± 6.1	5.2 ± 6.8
	C or less	4.9 ± 6.8	1.9 ± 4.8
	*p* value §	0.391	0.210

† Independent-samples Mann-Whitney U test

§ Independent-samples Kruskal-Wallis test

## Discussion

The current study evaluated the capabilities and accuracy of 91 junior dentists in diagnosing common oral mucosal changes and managing these changes during their internship year. A well-structured case-based educational lecture regarding common oral mucosal lesions with representative clinical pictures and appropriate management was created and conducted by an oral medicine consultant. A validated questionnaire composed of 20 cases was implemented for the assessment. Given the limited data on common oral lesions in Saudi Arabia, the lesions included in this study were chosen based on available local evidence [6, 7]. Dental interns’ knowledge significantly improved from 78% to 91% immediately after they attended the lecture. After two months of information retention assessment, there was no statistically significant difference observed in their diagnosis and management competencies. Gaballah and Kujan evaluated the effect of a half-day continuing education activity on 67 junior dentists’ abilities to recognize oral mucosal abnormalities. Their findings revealed an improvement in this capability from 55.1% to 74.8% [23]. Similarly, prior studies have demonstrated that continuing education can improve the ability of junior dentists to detect oral mucosal abnormalities [3, 24]. The improvement in the ability of junior dentists to diagnose oral lesions observed in the present study supports the need for regular educational programs involving oral lesion diagnosis and management to ensure a sustained level of clinical competency. Although interns with higher GPAs exhibited slightly smaller score gains, this pattern may reflect a ceiling effect, as these participants likely entered the study with stronger baseline knowledge. Importantly, the absence of statistically significant differences across GPA categories suggests that the educational intervention was equally effective for interns of varying academic performance levels. Our results revealed no significant sex difference, which is consistent with the findings of previous studies [10, 23]. However, Alharbi and Aboalela observed significantly greater male confidence in managing oral lesions in comparison to their female colleagues [10].

Oral lesions demonstrate diverse etiologies, and the recognition of the most common conditions is vital for early detection, accurate diagnosis, and appropriate management. This process can be challenging; however, ensuring effective patient care remains essential [8, 11, 12, 23]. A lack of general dentists’ knowledge regarding the diagnosis and management of these lesions can lead to excessive referrals. This scenario can consequently result in long waiting lists and represent a burden on the healthcare system, thus potentially causing delays in the diagnosis of serious oral conditions (including oral cancer) [1]. In a retrospective study conducted in Italy, 1,500 patient records from 2008 to 2014 were reviewed [25]. The most frequently reported lesions included aphthous lesions, traumatic ulcers, lesions caused by herpes simplex virus infections, geographic tongues, candidiasis, moriscatio buccarum, Fordyce granules, hyperkeratosis, and mucocele. Another study reported that the most common oral lesions encountered in clinical practice were recurrent aphthous stomatitis, herpes simplex virus-related lesions, oral lichen planus, mucocele, squamous cell papilloma, pyogenic granuloma, oral melanoma, amalgam tattoo, hairy tongue, and different types of oral candidiasis [26]. In a study of 598 dental patients in Tehran, developmental oral lesions were observed in 49.3% of cases, with Fordyce granules, fissured tongue, leukoedema, and hairy tongue being the most commonly observed lesions [27]. Al-Mobeeriek et al. reported that oral mucosal lesions were present in 15% of Saudi dental patients, with the highest prevalence observed among individuals aged 31–40 years; the most frequently identified lesions were Fordyce granules, leukoedema, traumatic lesions, and a range of tongue abnormalities such as fissured tongue and bifid tongue [6]. Similarly, additional studies from Saudi Arabia and the Gulf region have reported that Fordyce granules, leukoedema, traumatic ulcers, and aphthous ulcers constitute the predominant oral mucosal conditions in these population [2, 7, 8, 14]. The inclusion of these common entities ensured that the lecture content reflected actual clinical prevalence patterns, thereby enhancing its relevance for daily dental practice.

Dental students must complete several intensive courses focused on oral pathology and oral medicine before earning their degree. These courses include both theoretical lectures and practical clinical sessions involving actual patients. By the time they reach their senior years, dental students are expected to have acquired valuable knowledge that enables them to conduct complete medical and dental histories, along with comprehensive clinical examinations of orofacial structures. This knowledge should also empower them to identify and manage the most common oral conditions encountered in their clinical practice [10]. Due to this intensive education, their scores obtained before the lecture were relatively high, especially for the lesions that they had frequently observed during their undergraduate training. According to our results, a high percentage of the dental interns experienced no difficulties in diagnosing hairy tongue, geographic tongue, lingual varices and traumatic ulcers, due to the fact that these conditions are strongly emphasized in the curriculum of the KAU-dental school and are repeated at multiple levels throughout their undergraduate education. These findings reflect the strength of the KAU undergraduate oral medicine curriculum, wherein repeated clinical exposure across multiple years reinforces diagnostic proficiency. Nonetheless, the observed gains after the educational intervention emphasize the notion that periodic continuing education is essential to maintain and advance these competencies beyond graduation. In agreement with our findings, a previous study observed that compared with newly graduated GDPs, interns demonstrated the greatest ability to generate differential diagnoses and correctly classify oral lesions [28]. In a Saudi study investigating the knowledge of dental practitioners regarding oral mucosal lesions, compared with senior dentists, dental interns reported the highest proportion of correct responses [29]. These data can be explained by the fact that interns are more likely to retain knowledge compared to GDPs, who may experience a decline in foundational knowledge over time. Furthermore, interns are generally more engaged in focused study efforts due to the rigorous preparation required for licensing examinations. In contrast, a study from Turkey reported that 85% of GDPs exhibited limited diagnostic ability for oral mucosal lesions [30]. In the present study, the initial diagnostic competency of the interns (81.5%) exceeded their management competency (74.5%), thus reflecting that management skills may require further strengthening through practical experience and continuing education. The educational lecture significantly improved both of the aforementioned competencies (94% and 88%, respectively). Recent investigations have similarly demonstrated that brief, targeted educational interventions can significantly improve diagnostic accuracy and decision-making among dental and medical trainees [3, 23]. These studies align with the present findings and highlight the continuing importance of structured, evidence-based reinforcement in oral medicine education.

An interesting finding in this study was that interns in our cohort demonstrated relatively low prescribing scores (46.1%), which may be attributed to the limited emphasis on pharmacotherapeutics and prescription writing within undergraduate dental curricula. In most institutions, prescribing responsibilities are typically undertaken by oral medicine consultants, thus offering students minimal hands-on exposure to this skill during training [31]. Although prescribing performance significantly improved immediately after the educational intervention, a marked decline was observed at the two-month follow-up. This pattern suggests that although the diagnostic recognition of oral lesions tends to remain stable, the ability to select and prescribe appropriate pharmacological management is more vulnerable to decay over time [32, 33]. Such findings underscore the need for ongoing reinforcement and experiential learning through practical, case-based pharmacotherapy sessions to sustain prescribing competence and clinical decision-making skills among dental interns [34].

It is essential to acknowledge the limitations of this study design. First, the evaluation solely relied on a knowledge test, which primarily assesses factual recall. Although foundational knowledge is crucial, it does not necessarily translate directly to clinical competency. Future studies could incorporate practical assessments, such as case studies or simulations, in order to evaluate the ability of interns to apply the learned knowledge in clinical scenarios on real patients. Second, although retention was examined, the long-term retention of knowledge was not assessed. Didactic lectures alone may not be sufficient for long-term knowledge retention. The implementation of spaced repetition techniques or the incorporation of follow-up sessions could enhance knowledge consolidation. Further studies investigating the impact of continuous educational activities on the knowledge of dentists with different levels of experience are recommended. Another limitation is that a sample size calculation was not performed before data collection, which may have affected the statistical power to detect smaller effects. Moreover, the pilot validation included only three participants, thereby limiting the generalizability of the reliability results, despite the excellent kappa value that was obtained. Future studies should include larger samples and a pre-study power analysis to ensure sufficient representativeness. Moreover, the study’s short follow-up period due to scheduling conflicts, as well as the attrition rate (43.5%), single-institution sample, lack of a control group, and absence of power analysis, may limit generalizability. The limited two-month follow-up likely reflects short-term gains rather than enduring knowledge retention. Additionally, the attrition of 43.5% may have introduced selection bias, as more motivated participants were more likely to complete all of the stages. In addition, potential testing effects due to the use of similar case images should be acknowledged. Future research should include multi-institutional designs and extended retention assessments.

This study aimed to develop a compact online lecture that can significantly increase knowledge and awareness regarding the most common oral conditions that dentists may encounter through their professional careers. The implementation of such a continuing education activity is recommended to improve the ability of dentists to assess the severity of oral lesions and determine appropriate management strategies, thus leading to a better overall experience for the patients.

## Supplementary Information

Supplementary Material 1.

## Data Availability

The datasets used and/or analyzed during the current study are available from the corresponding author upon reasonable request.

## Abbreviations

MCQs: multiple-choice questions
GPA: grade point average
GDPs: general dental practitioners
KAU: King Abdulaziz University
