# Supplementary material for: Improving diagnostic and management competencies for common orofacial conditions among new dental graduates: the effect of an educational intervention
Source: BMC Oral Health. 2026 Jan 12;26:261. doi: 10.1186/s12903-026-07662-7 (PMC12888301; doi:10.1186/s12903-026-07662-7)
Supplement: Supplementary file 1 — Supplementary Material 1. [file 12903_2026_7662_MOESM1_ESM.pdf]

Diagnostic and Management Skills for Common Orofacial Conditions Among Recent Dental Graduates

Dear Intern,

You are invited to participate in this study aiming to address the knowledge of dental fresh graduates in the management of the common oral lesions.
Completing the questionnaire will require about five minutes and this will help us to achieve the goals of this research.
Your participation in this study is entirely voluntary and your identity will remain completely anonymous and all information you provide will be treated with confidentiality and will be used for research purposes only. By completing this questionnaire, you are giving your consent to participate in this study and to use the corresponding anonymized data for publication.
We appreciate your time and participation and thank you for your cooperation

* Indicates required question

1. Do you consent in participating in the study and to use the submitted anonymized data for publication.?

   Mark only one oval.

   ◯ Yes
   ◯ No

2. What gender are you ? *

   Mark only one oval.

   ◯ Male
   ◯ Female

3. GPA

   ____

4. A 40-year-old male patient who smokes cigarettes daily for the last 20 years, presented to the clinic complaining of irritation in the palate. What is the most probable diagnosis?

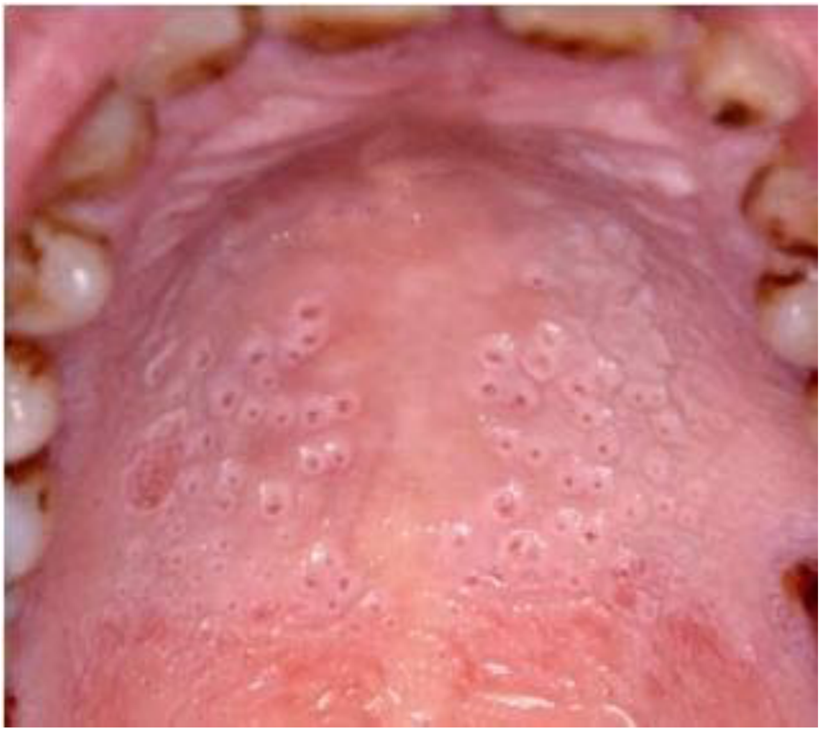

Mark only one oval.

- Denture stomatitis
- Nicotinic stomatitis
- Erythematous candidiasis
- Fordyce granules
- I don’t know

5. How would you manage this case? *

Mark only one oval.

- Reassure patient
- Smoking cessation
- Betamethasone dipropionate gel
- Lidocaine spray
- I don’t know

6. 28-year-old female patient complaining of a painful ulcer in her lip. She reported several recurrence of similar ulcers over the past years. She is otherwise healthy. What is the most probable diagnosis?

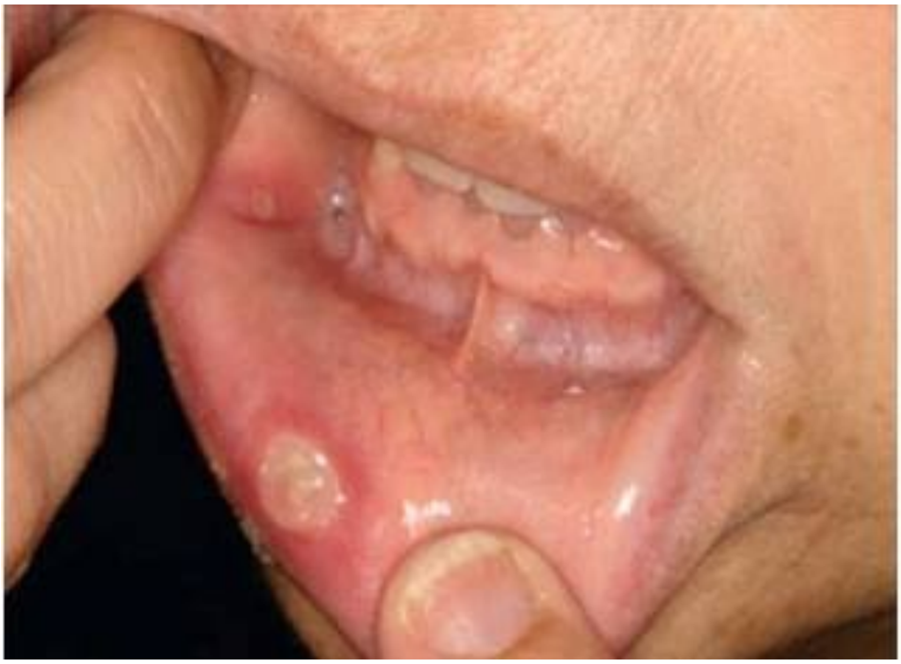

Mark only one oval.

- Traumatic ulcer
- Primary herpetic gingivostomatitis
- Lichen planus
- Recurrent apthous stomatitis
- I don't know

7. How would you manage this case? *

Mark only one oval.

- Reassure patient
- lidocaine spray
- Betamethasone dipropionate gel
- Excision
- I don't know

8. A 60-year-old female patient came for regular checkup. Upon intraoral examination, you found this lesion on the buccal mucosa. The patient reported mild to moderate pain in association with this lesion. What is the most probable diagnosis?

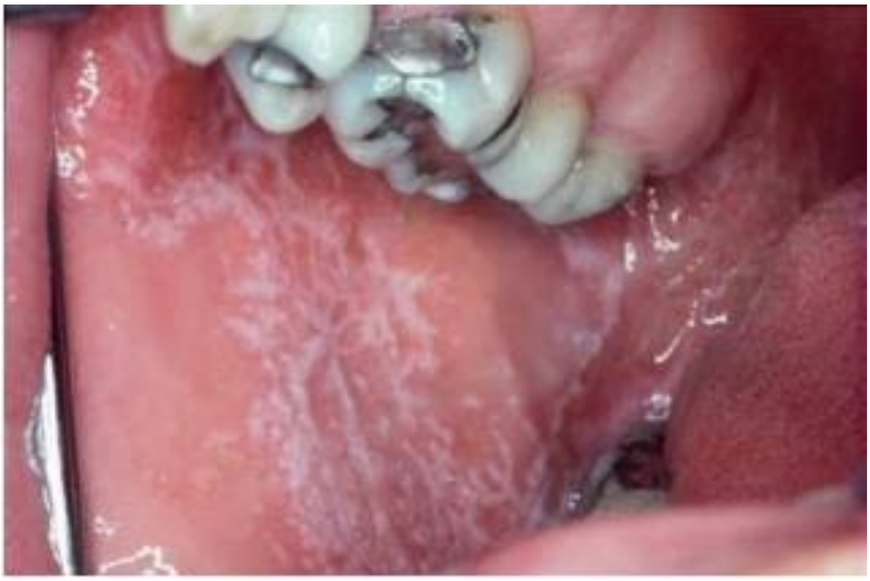

Mark only one oval.

- Oral lichen planus
- Frictional keratosis
- Oral candidiasis
- Leukodema
- I don't know

9. How would you manage this case? *

Mark only one oval.

- lidocaine spray
- Betamethasone dipropionate gel
- Excision
- Nystatin oral suspension
- I don't know

10. A 15-year-old female patient presented to the clinic complaining from soreness on the lip. What is the most probable diagnosis?

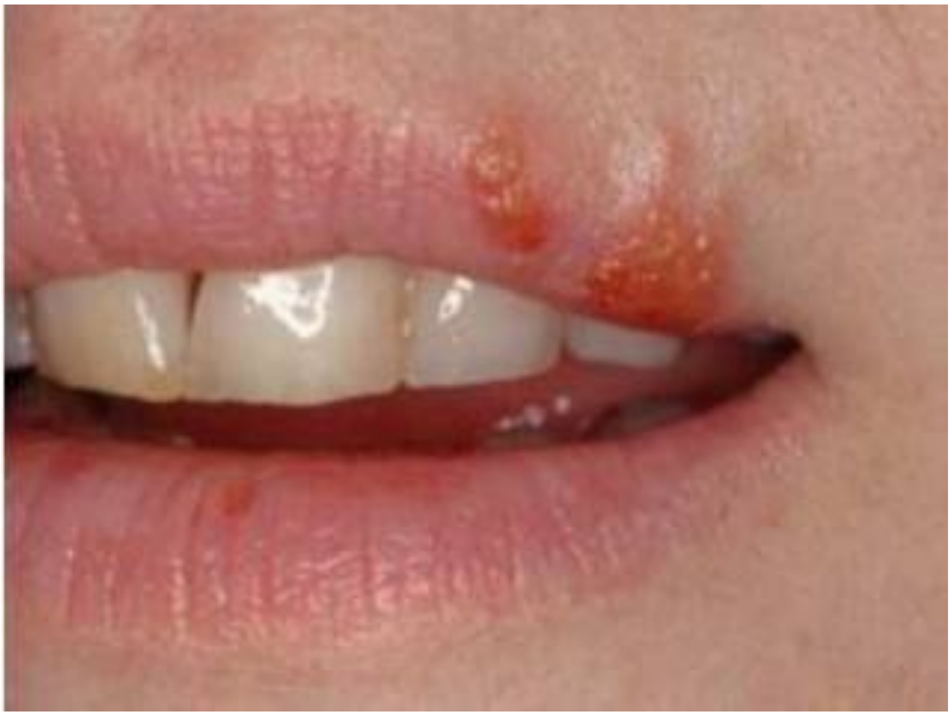

Mark only one oval.

- ◯ Recurrent apthous stomatitis
- ◯ Herpes labialis
- ◯ Lichen planus
- ◯ Primary herpetic gingivostomatitis
- ◯ I don't know

11. How would you manage this case? *

Mark only one oval.

- ◯ Betamethasone dipropionate gel
- ◯ Lidocaine Hydrochloride gel
- ◯ Acyclovir cream
- ◯ Miconazole Nitrate
- ◯ I don't know

12. A 14-year-old patient presented with short tongue and reported difficulty in speech. What i the most likely diagnosis?

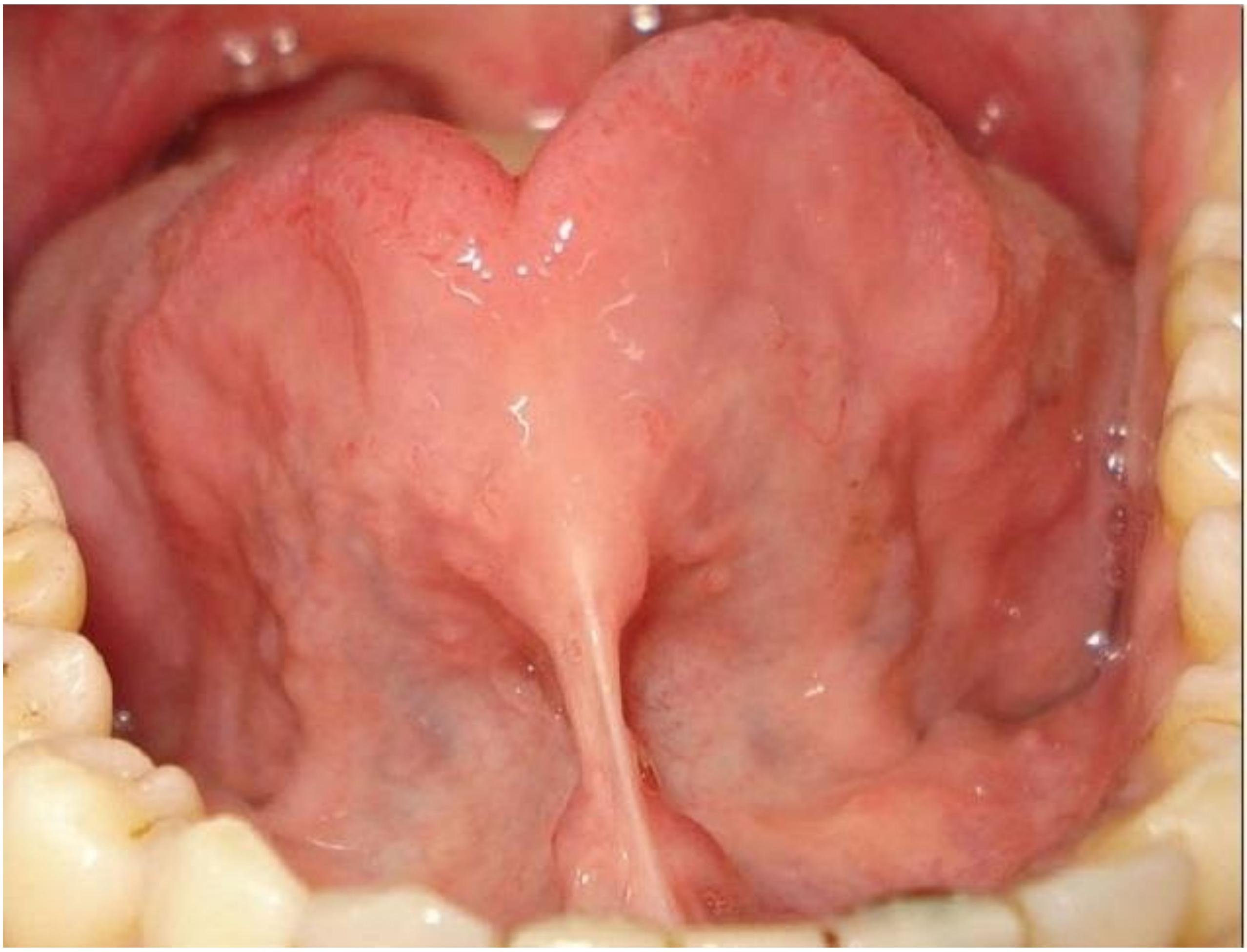

Mark only one oval.

- Bifid tongue
- Fibrosis
- Ankyloglossia
- Ranula
- I don’t know

13. How would you manage this case? *

Mark only one oval.

- Reassurance only
- Lingual frenectomy
- Lidocaine hydrochloride gel
- Referral to speech therapist only
- I don’t know

14. A 20-year-old female patient presented to the clinic with indentations on her tongue. What is the most probable diagnosis?

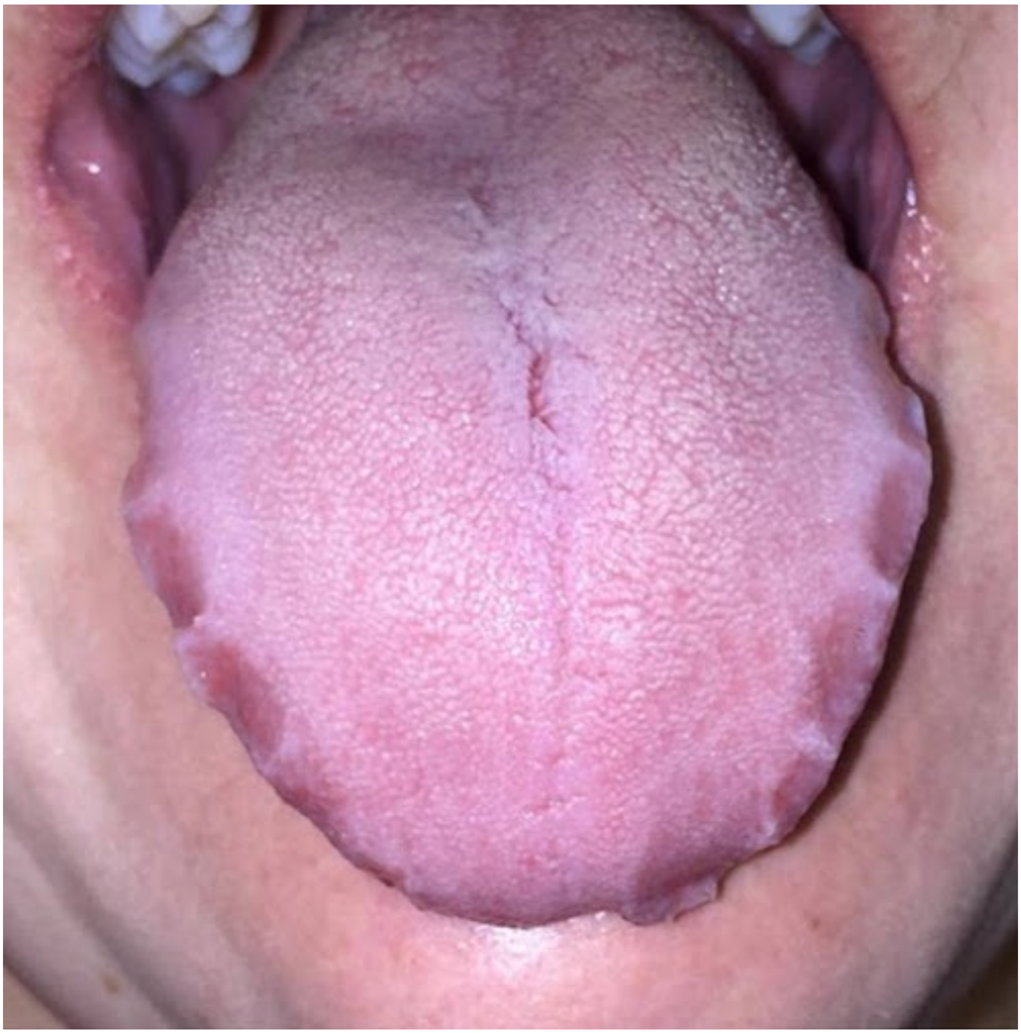

Mark only one oval.

- ( ) Tongue tie
- ( ) Scalloped tongue
- ( ) bifid tongue
- ( ) I don’t know

15. How would you manage this case? *

Mark only one oval.

- No treatment
- Betamethasone dipropionate gel
- Splint
- Orabase
- I don’t know

16. A 30-year-old male patient came with a painless nodule in the lower lip. What’s the probable diagnosis?

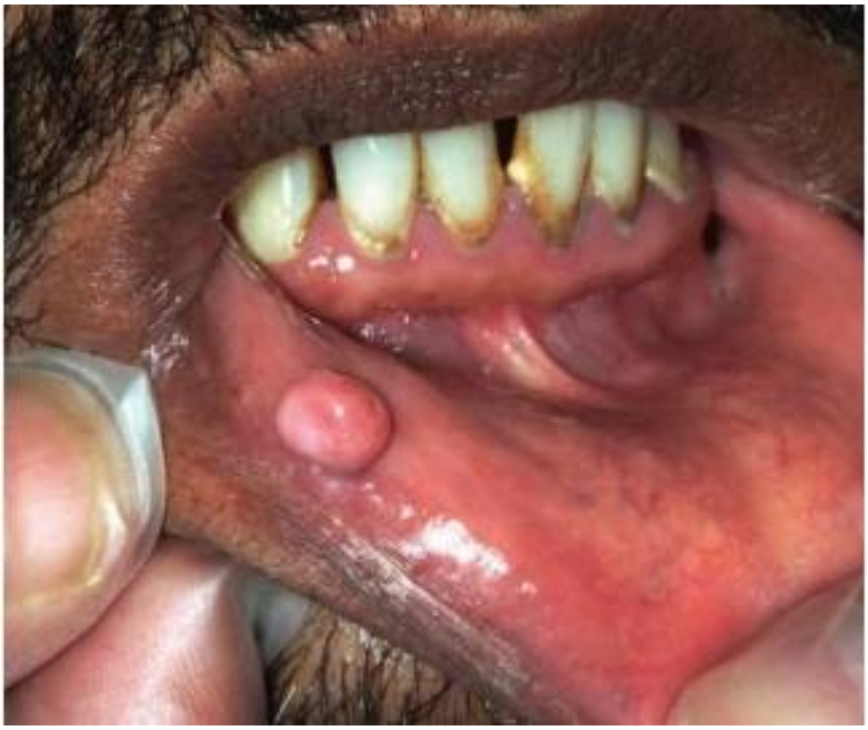

Mark only one oval.

- lipoma
- Irritating fibroma
- Focal fibrous hyperplasia
- pyogenic granuloma
- I don’t know

17. How would you manage this case? *

Mark only one oval.

- Topical anesthetic
- Surgical excision
- Betamethasone dipropionate gel
- No treatment
- I don’t know

18. A female patient attended the clinic complaining of a lesion related to the anterior two-thirc of her tongue with soreness associated with spicy food. What’s the most probable diagnosis?

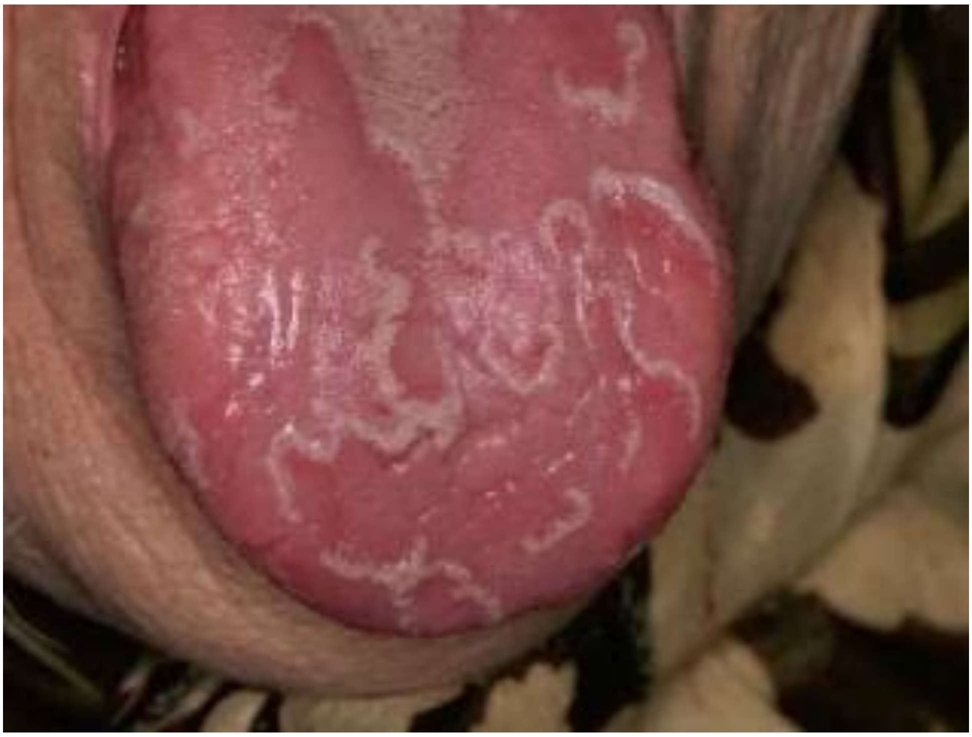

Mark only one oval.

- Pseudomembranous candidiasis
- Hairy tongue
- Fissured tongue
- Geographic tongue
- I don’t know

19. How would you manage this case? *

Mark only one oval.

- Betamethasone dipropionate gel
- Amoxicillin
- Lidocaine Hydrochloride gel
- Chlorohexidine mouthwash
- I don’t know

20. A 60-year-female patient came for a regular check-up. Upon clinical examination, you found dilated tortuous veins in the ventral side of the tongue. What is the most probable diagnosis ?

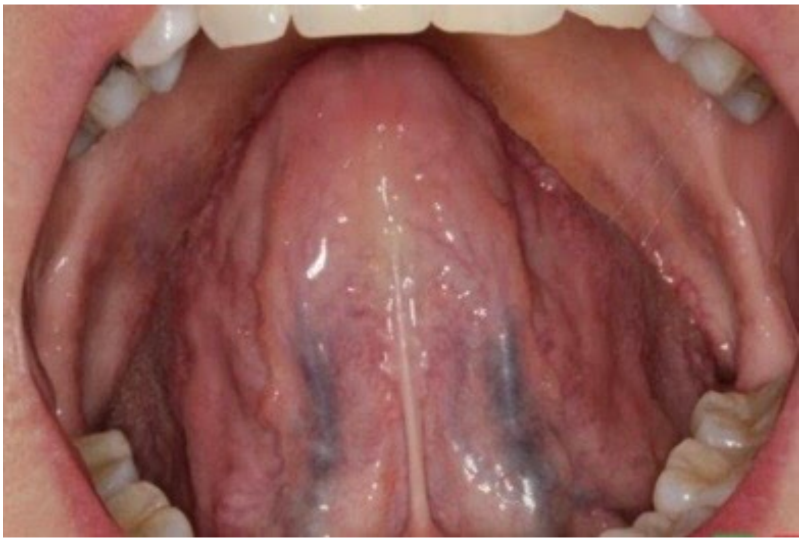

Mark only one oval.

- Lingual varices
- Plical fimbrata
- frictional keratosis
- I don’t know

21. How would you manage this case? *

Mark only one oval.

- Reassurance
- Betamethasone dipropionate gel 0.05%
- Surgery
- Follow up
- I don’t know

22. A 25-year-old female patient complaining of swelling in the lower lip. The swelling was painless and cystic on palpation, it enlarged when smelling food. Whit is the most probable diagnosis?

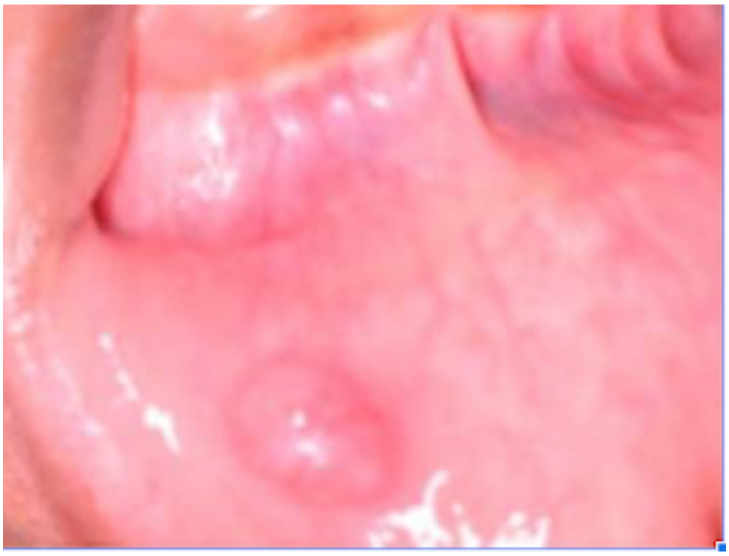

Mark only one oval.

- Mucocele
- Ranula
- Lipoma
- Fibroma
- I don’t know

23. How would you manage this case? *

Mark only one oval.

- Surgical removal
- Betamethasone dipropionate gel
- No treatment
- Orabase
- I don't know

24. A 30-year-old pregnant female attended the clinic concerned with gingival enlargement. What is the most probable diagnosis ?

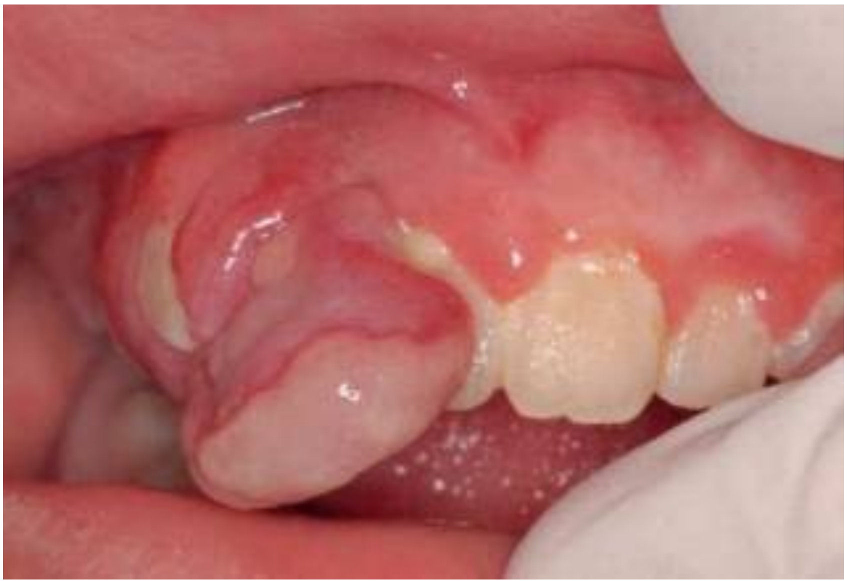

Mark only one oval.

- Pyogenic granuloma
- fibroma
- lipoma
- peripheral giant cell granuloma
- I don't know

25. How would you manage this case? *

Mark only one oval.

- ⬭ Surgical excision
- ⬭ Betamethasone dipropionate gel
- ⬭ Lidocaine Hydrochloride gel
- ⬭ Reassure the patient
- ⬭ I don’t know

26. A 29-year-old female patient came for a regular check-up. Upon examination, you found a loss of papilla in the dorsum of the tongue, the patient occasionally feels soreness. She is on a diet to loss weight. What is the most probable diagnosis ?

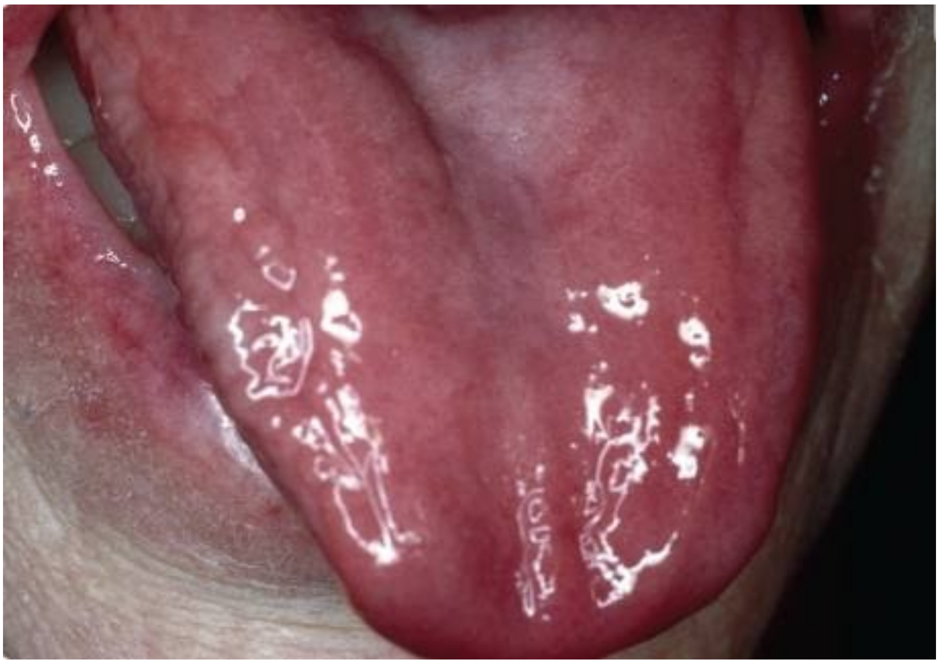

Mark only one oval.

- ⬭ atrophic glossitis
- ⬭ scalloped tongue
- ⬭ erythamatous candidiasis
- ⬭ fissured tongue
- ⬭ I don’t know

27. How would you manage this case? *

Mark only one oval.

- Amoxicillin
- Acyclovir cream
- Order CBC, Vitamin B12 and ferritin serum level and refer to physician if needed
- Reassurance
- I don't know

28. 40-year-old male patient came to the clinic for regular check-up. White lesion on the bucca mucosa found and it disappears when the mucosa is stretched. What is the diagnosis ?

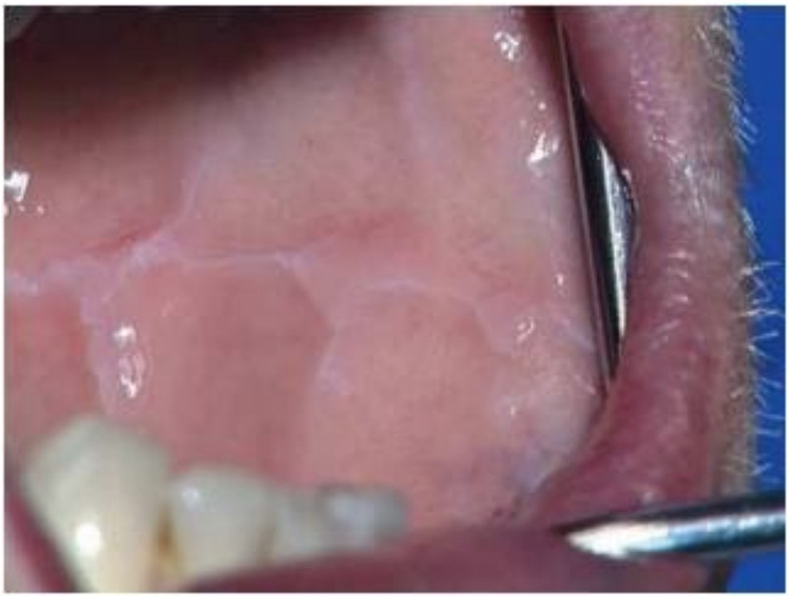

Mark only one oval.

- Leukoedema
- Leukoplakia
- Morsicatio buccarum
- Oral lichen planus
- I don't know

29. How would you manage this case? *

Mark only one oval.

- Orabase
- Amoxicillin
- Reassure the patient
- I don't know

30. An 18-year-old male patient came to the clinic complaining of pain in his tongue, patient had a broken sharp amalgam restoration. What is the most probable diagnosis?

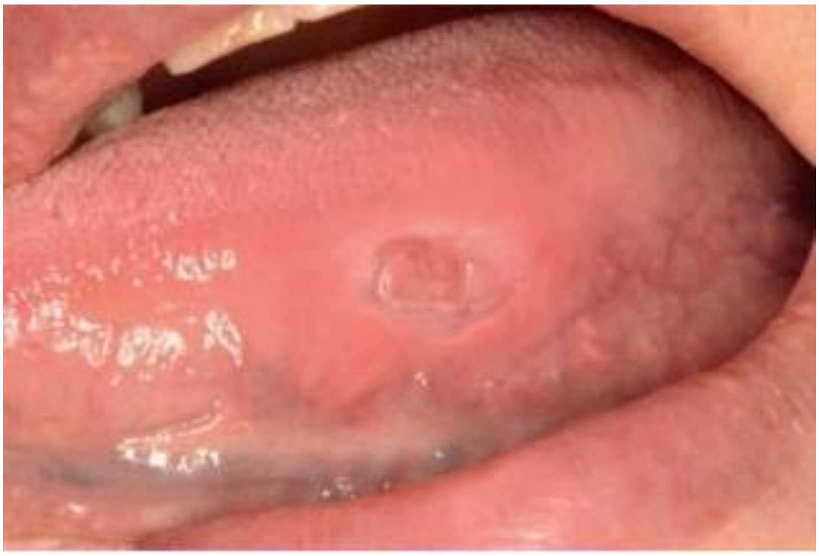

Mark only one oval.

- Recurrent aphthous stomatitis
- Primary herpetic gingivostomatitis
- Traumatic ulcer
- Squamous cell carcinoma
- I don't know

31. How would you manage this case? *

Mark only one oval.

- ⬭ Betamethasone dipropionate gel
- ⬭ Amoxicillin
- ⬭ Smoothening of sharp edges
- ⬭ Lidocaine Hydrochloride gel
- ⬭ I don't know

32. A24 year old male patient came to the clinic complaining of the appearance of her tongue. What is the most probable diagnosis ?

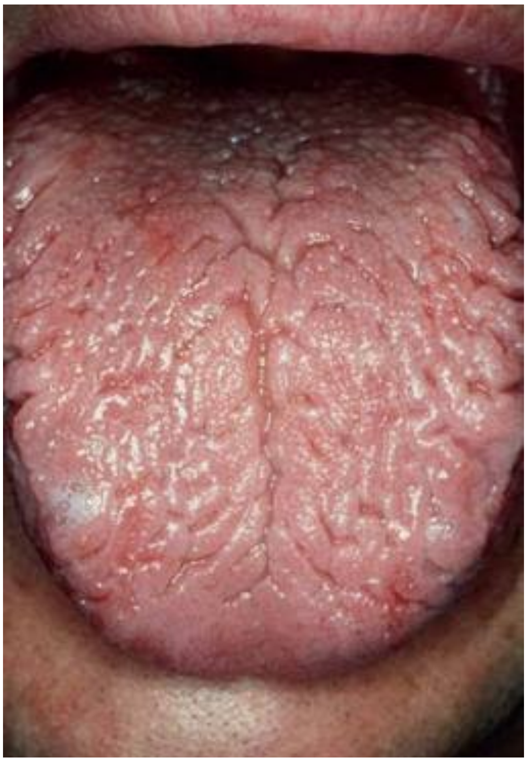

Mark only one oval.

- ⬭ Fissure tongue
- ⬭ Erythematous candidosis
- ⬭ Geographic tongue
- ⬭ Hairy tongue
- ⬭ I don't know

33. How would you manage this case? *

Mark only one oval.

- Nystatin oral suspension
- Betamethasone dipropionate gel
- Acyclovir cream
- Reassure the patient
- I don’t know

34. 40-year-old female patient presented to clinic to restore various teeth during clinical examination you saw this structure . What is the most probable diagnosis?

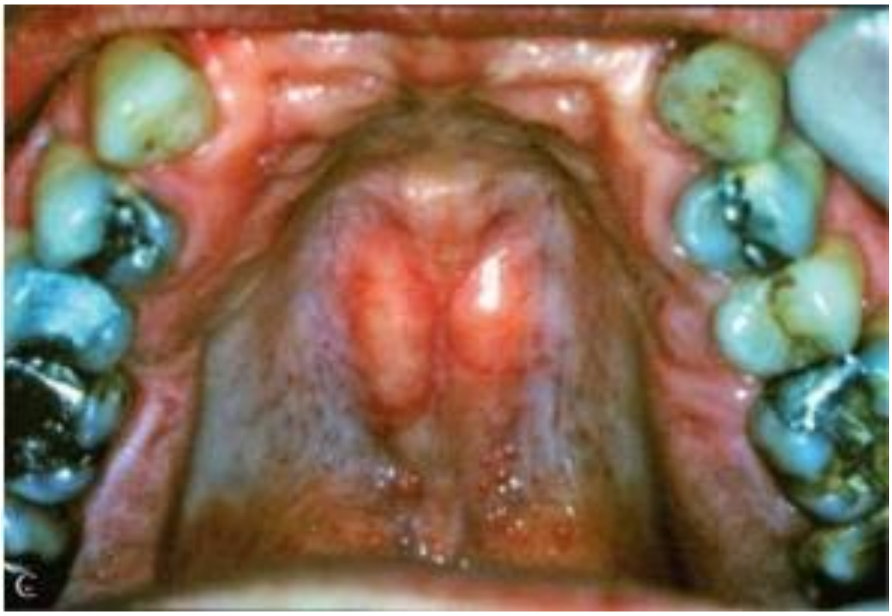

Mark only one oval.

- Leukoplakia
- Torous paltinus
- Pleomorphic adenoma
- oral lichen planus
- I don’t know

35. How would you manage this case? *

Mark only one oval.

- Surgical excision
- Acyclovir cream
- Betamethasone dipropionate gel
- Reassure the patient
- I don’t know

36. 38 year old male patient complaining of white roughness in his left cheek. The patient says he bites his cheeks. What is the most probable diagnosis ?

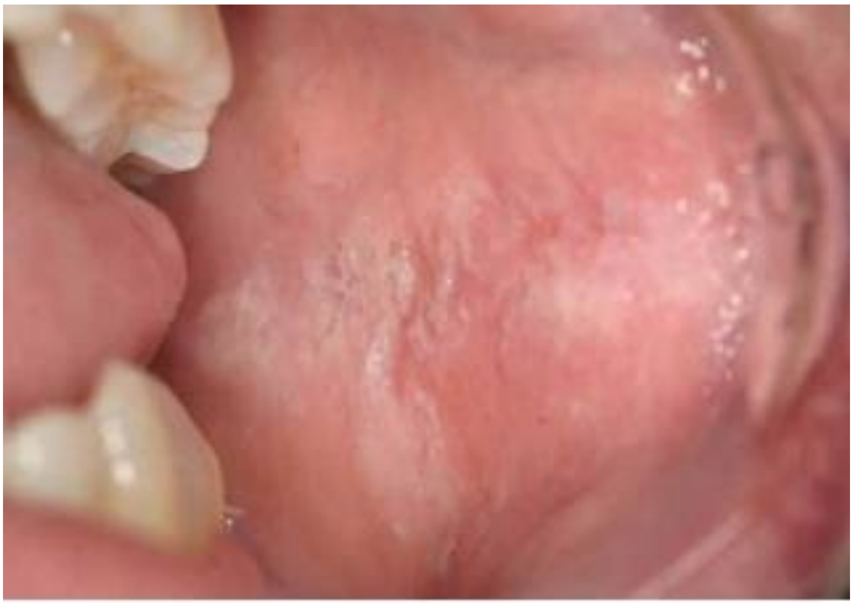

Mark only one oval.

- Lina alba
- Geographic tongue
- Oral lichen planus
- Morsicatio buccarum
- I don’t know

37. How would you manage this case? *

Mark only one oval.

- Tongue brushing
- Acyclovir cream
- Nystatin
- Patient education
- I don’t know

38. 50 year old female patient presented to the clinic complaining of her tongue. She smokes two packs a day. What is the most probable diagnosis ?

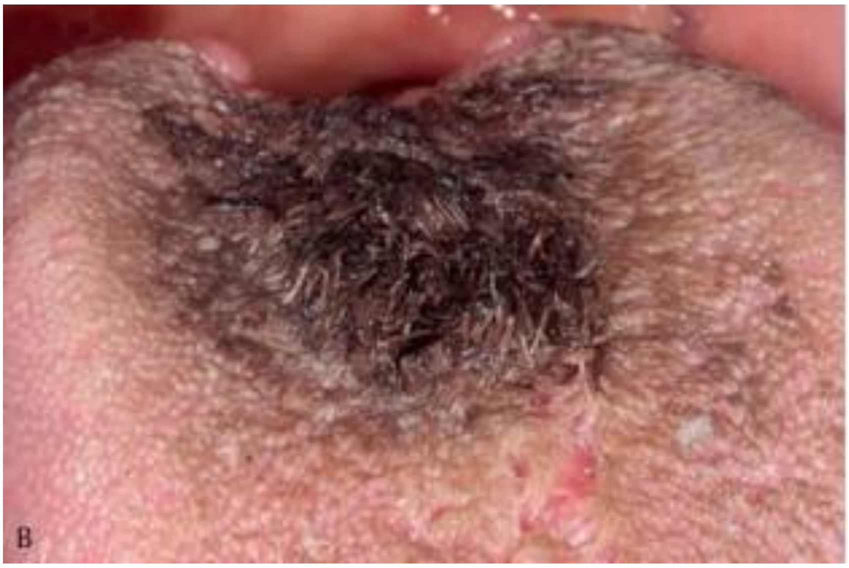

Mark only one oval.

- Leukoplakia
- Hairy tongue
- Coated tongue
- Geographic tongue
- I don’t know

39. How would you manage this case? *

Mark only one oval.

- Tongue brushing
- Acyclovir cream
- Nystatin oral suspension
- Miconazole Nitrate gel
- I don’t know

40. A 45 year old male patient came to the clinic for regular check up, upon clinical examination you found diffused discoloration. What is the most probable diagnosis ?

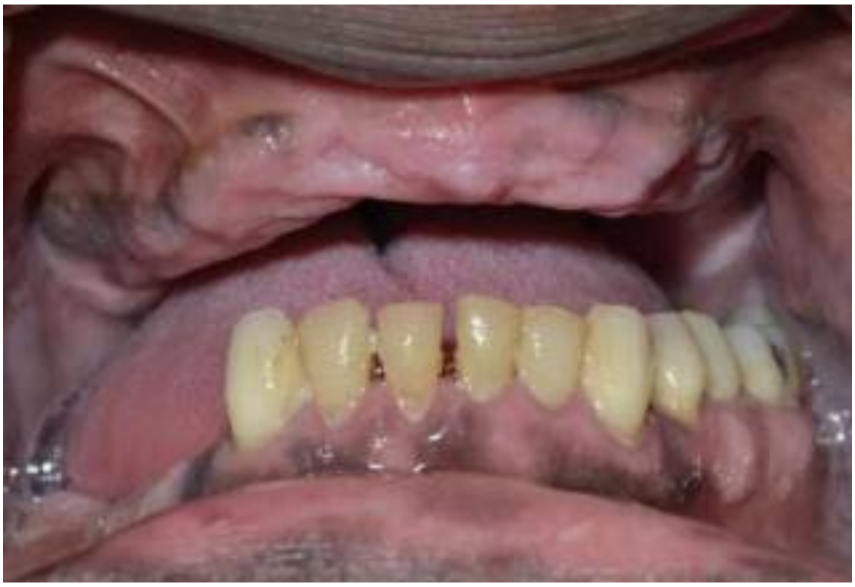

Mark only one oval.

- Heavy Metal pigmentation
- Drug-Induced pigmentation
- Smoker melanosis
- Physiologic pigmentation
- I don’t know

41. How would you manage this case? *

Mark only one oval.

- Betamethasone dipropionate gel
- Acyclovir cream
- Discontinuation of the drug
- Reassure the patient
- I don’t know

42. A 30 year old male patient came to the clinic concerned of multiple nodules on his buccal mucosa. What is the most probable diagnosis ?

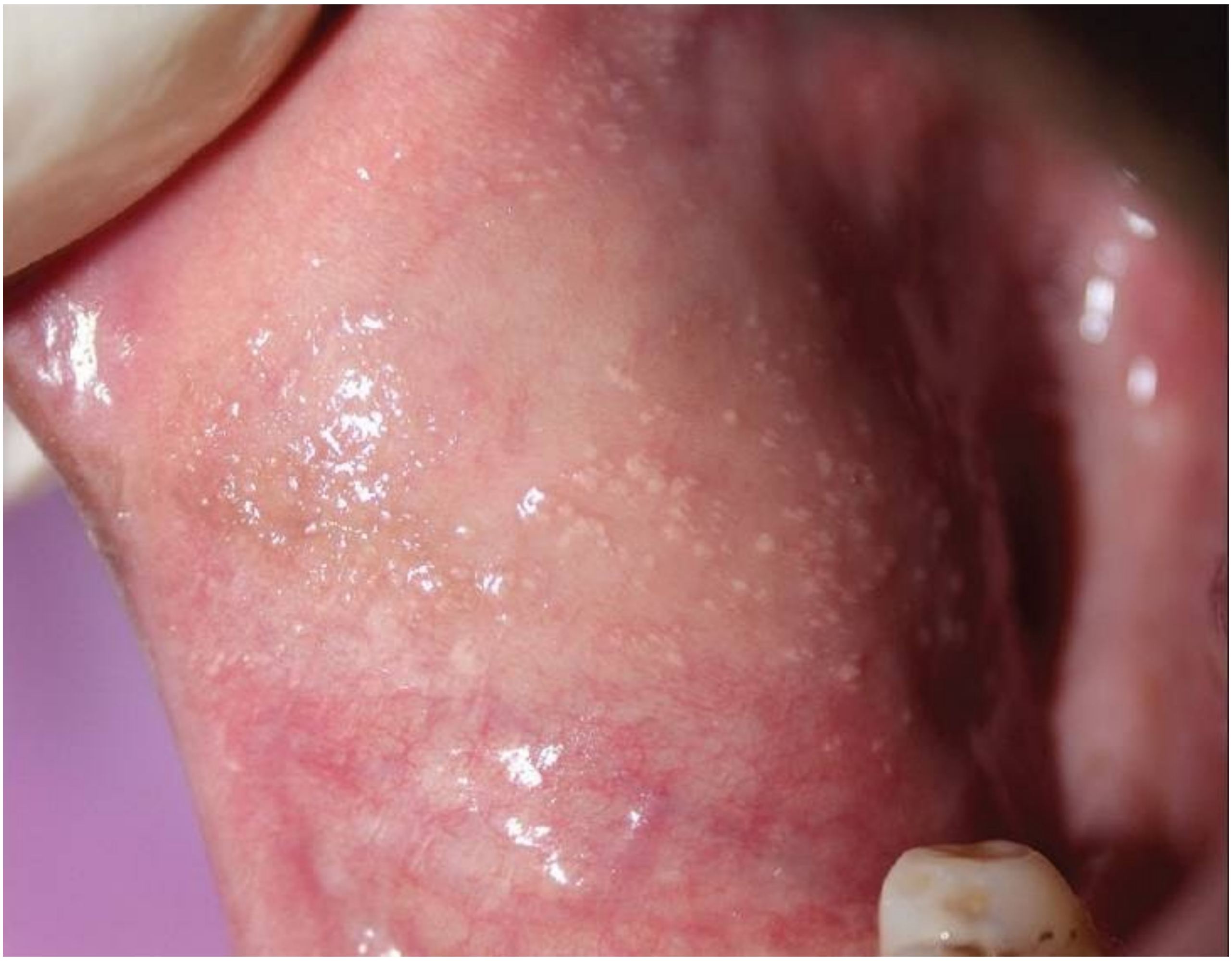

Mark only one oval.

- Pyogenic granuloma
- frictional keratosis
- Lipoma
- Fordyce granules
- I don’t know

43. How would you manage this case? *

Mark only one oval.

- Miconazole Nitrate gel
- Betamethasone dipropionate gel
- Surgical incision
- Reassure the patient
- I don’t know

This content is neither created nor endorsed by Google.

Google Forms
